# Detecting Airborne Pollen Using an Automatic, Real-Time Monitoring System: Evidence from Two Sites

**DOI:** 10.3390/ijerph19042471

**Published:** 2022-02-21

**Authors:** Maria Pilar Plaza, Franziska Kolek, Vivien Leier-Wirtz, Jens Otto Brunner, Claudia Traidl-Hoffmann, Athanasios Damialis

**Affiliations:** 1 Environmental Medicine, Faculty of Medicine, University of Augsburg, 86156 Augsburg, Germany; maria.plaza@tum.de (M.P.P.); franziska.kolek@tum.de (F.K.); vivien.leier-wirtz@tum.de (V.L.-W.); claudia.traidl-hoffmann@tum.de (C.T.-H.); 2 Institute of Environmental Medicine, Helmholtz Center Munich—German Research Center for Environmental Health, 86156 Augsburg, Germany; 3 Health Care Operations/Health Information Management, Faculty of Business and Economics, Faculty of Medicine, University of Augsburg, 86159 Augsburg, Germany; jens.brunner@uni-a.de; 4 Christine Kühne—Center for Allergy Research and Education (CK-CARE), 7265 Davos, Switzerland; 5 Department of Ecology, School of Biology, Faculty of Sciences, Aristotle University of Thessaloniki, 54124 Thessaloniki, Greece

**Keywords:** aerobiology, automatic pollen monitoring, environmental health services, real-time monitoring, sensitivity analysis

## Abstract

Airborne pollen monitoring has been an arduous task, making ecological applications and allergy management virtually disconnected from everyday practice. Over the last decade, intensive research has been conducted worldwide to automate this task and to obtain real-time measurements. The aim of this study was to evaluate such an automated biomonitoring system vs. the conventional ‘gold-standard’ Hirst-type technique, attempting to assess which may more accurately provide the genuine exposure to airborne pollen. Airborne pollen was monitored in Augsburg since 2015 with two different methods, a novel automatic Bio-Aerosol Analyser, and with the conventional 7-day recording Hirst-type volumetric trap, in two different sites. The reliability, performance, accuracy, and comparability of the BAA500 Pollen Monitor (PoMo) vs. the conventional device were investigated, by use of approximately 2.5 million particles sampled during the study period. The observations made by the automated PoMo showed an average accuracy of approximately 85%. However, it also exhibited reliability problems, with information gaps within the main pollen season of between 17 to 19 days. The PoMo automated algorithm had identification issues, mainly confusing the taxa of *Populus*, *Salix* and *Tilia*. Hirst-type measurements consistently exhibited lower pollen abundances (median of annual pollen integral: 2080), however, seasonal traits were more comparable, with the PoMo pollen season starting slightly later (median: 3 days), peaking later (median: 5 days) but also ending later (median: 14 days). Daily pollen concentrations reported by Hirst-type traps vs. PoMo were significantly, but not closely, correlated (*r* = 0.53–0.55), even after manual classification. Automatic pollen monitoring has already shown signs of efficiency and accuracy, despite its young age; here it is suggested that automatic pollen monitoring systems may be more effective in capturing a larger proportion of the airborne pollen diversity. Even though reliability issues still exist, we expect that this new generation of automated bioaerosol monitoring will eventually change the aerobiological era, as known for almost 70 years now.

## 1. Introduction

Air quality and composition significantly influence human health, with specific constituents such as bioaerosols (i.e., airborne pollen and fungal spores) comprising a dire environmental agent often held responsible for a variety of allergic diseases [1,2]. Hence, there have been approximately 400 monitoring networks across the world that have traditionally measured the abundance and occurrence of pollen and fungal spores [3], mainly aiming to inform allergic individuals and medical practitioners. While the above has been taking place for several decades, the methodological approach of Hirst-type volumetric sampling is practically 70 years old [4]. On the other hand, it is currently considered as the ‘gold-standard’ of biomonitoring, with the occurring pollen and spore concentration datasets comprising some of the longest and most valuable biological time-series in the world. Such data have repeatedly contributed identification long-term trends in abundances, alterations in biodiversity of fungi and plants, and quantification impacts of climate change [5,6]. During the recent COVID-19 pandemic, the importance of such monitoring networks has been highlighted even more, given that higher airborne pollen concentrations have been proven to be positively correlated with higher numbers of SARS-CoV-2 infections [7].

Despite the abovementioned increased need for longer time-series, higher-resolution real-time data, up to now biomonitoring of airborne pollen and fungal spores is still conducted with labour intensive, temporally low-resolution measurements, which usually deliver measurements with a delay of at least a week. Nonetheless, it is well known, e.g., as in [8], that there is an immediate, and the strongest, effect of airborne pollen on the same day as the actual exposure, when the most intense respiratory symptoms are observed, as well as the strongest immune reactions. Any delay in the dissemination of pollen exposure information beyond a day is, as a matter of fact, considered obsolete in terms of practical allergy management and medical treatment. Concentration–response associations are essential to understand when and how much pollen concentration will cause significant health risks to the population, and previous evidence suggests that these interrelations are complex, non-linear and taxon-specific [9,10]. This variability highlights the necessity for research across different pollen taxa and locations to provide consistent and locally significant evidence on the clinical relevance of pollen data and forecasts.

While the techniques followed in [4], to their best of their ability, attempt to simulate a normal respiratory function of a human in calm conditions (10 L min^−1^), this might be far from the real-life situation: in at least two thirds of our lives we work, walk, converse, laugh, eat or exercise, which altogether immediately increase this rate to much higher levels. Moreover, this kind of traditional low-volume sampling technique may reduce the accuracy of concentration estimates for rarer particles. This practically means that we may still be far from the assessment of the genuine exposome and even farther from the definition of concentration thresholds for pollen and spores, the quantification of the allergic (among others) reactome and symptomatology, and the optimal management of bioaerosol-associated diseases.

Having said this, the most consistent obstacle to the technological advancement of bioaerosol research and biomonitoring techniques has been, in contrast to the detection of chemical pollutants, the lack of continuous public funding (if at all). For this reason, even though bioaerosol time-series are some of the longest modern biological data-series worldwide, they are usually not open-access, as they reflect the effort and expenses of research institutes, universities and of private initiatives. Therefore, an urgent switch is needed towards faster, online and more accurate reporting of airborne pollen and spore concentrations, a requirement repeatedly noted as a hot topic in the aerobiological community [11,12].

Recent efforts have focused on developing automated sampling devices in real time, which have been developed on the borderline of the sciences of microbiology, engineering and informatics. Such approaches have already improved the efficiency of particle capturing and recognition, as well as data flow and dissemination, with results obtained allowing for larger-scale ecological interpretations. These new techniques refer to (among others) air-flow cytometry, such as the Yamatronics KH-3000 [13], Plair PA-300 [14,15], Pollen Sense^TM^ [16] or the WIBS sensor [17]. DNA metabarcoding using trnL and nrITS2 have also shown highly improved taxonomic resolution for pollen from aerobiological samples [18,19], even though not at a (near-)real-time temporal resolution. Still, there is a huge gap in the application of automation techniques regarding other bioaerosols, i.e., fungi [20] or bacteria [21], as well as development of such methods in indoor environments, as has been traditionally elaborated [20,21].

Apart from these recent advances in biomonitoring systems, palynology (including aerobiology) traditionally relies on image-based identification of particles [22]. Hence, automating this process has attracted attention for more than a decade now and is still one of the central pillars of technological progress [3,23]. The high-throughput flow that, in any case, improved the efficiency of these devices compared to the conventional Hirst-type, has been complemented by automated microscopy for particle identification per pollen/spore type assisted with the use of image recognition algorithms [24,25,26,27]. If, to the above, advanced deep learning methods are integrated, the results already seem outstanding [28,29,30,31].

The first and most prevalent of such biomonitoring systems, based on automatic image recognition, is the BAA500 (Hund GmbH), established and running fully operationally in Bavaria, Germany [32], which has reported significant preliminary performance results when using pollen recognition algorithms [26]. It has been reported that more than 90% of the pollen identified was correctly recognized [26,33]. However, under such rapid technological advancements, there are some common pitfalls at the beginning, such as reliability of the devices after longer-term monitoring and experimental repetitions, comparability of techniques and datasets, and reporting biases. 

In this paper, we use a unique dataset, the largest of its kind to the best of our knowledge, in terms of manually identified pollen images, as well as the total diversity of pollen taxa that are processed and included in the monitoring system evaluation. Here, we present the results of two different BAA500 (PoMo) devices. We report the reliability, performance, accuracy and comparability of the biomonitoring system, also comparing the commercial image library against the improved, manually classified version, as well as comparing against the ‘gold standard’ Hirst-type pollen measurements. The ultimate aim was to evaluate if the automatic, near-real-time biomonitoring system differs from the conventional one, and, consequently, to identify the pros and cons in order to contribute to the optimal and operational development of this novel technique.

## 2. Materials and Methods

Airborne pollen has been monitored by the use of conventional and novel sampling devices, co-located aside each other. The biomonitoring sites were located in Augsburg, Germany, in:

(A) LFU (Landesamt für Umwelt) to the southeast of Augsburg city (48°19′33.6″ N, 10°54′10.8″ E) at 1.5 m above ground level (agl) since May 2015.
(B) IEM (Institute of Environmental Medicine) to the northwest of Augsburg city (48°23′04.15″ N, 10°50′35.95″ E) at 4 m agl since August 2017.

Both sites were located in a semi-urban environment characterized by a diverse urban flat landscape including buildings, vegetation and water surfaces, without any different and important source of pollen immediately close to the samplers.

The conventional sampling technique refers to the ‘gold-standard’ Hirst-type volumetric biomonitoring with a continuous flow of 0.6 m^3^/h (calibrated every week) as described in detail and recommended by the European Aerobiology Society (EAS) [34]. All pollen grains were identified by expert aerobiologists under a light microscope at ×400 magnification. Concentrations were expressed as the daily average number of pollen grains per cubic meter of air.

The novel, automatic device refers to the Bio-Aerosol Analyser BAA 500 (Pollen Monitor—PoMo). The pollen grains are extracted with an airflow of max. 6 m^3^/h with a special virtual impactor. The sampling intervals of the pollen monitor can be adjusted by the operator and were collected during 3 hourly periods (3 m^3^ during each sampling period), 24 m^3^ of air every day. Particles of sizes between 5 μm and 100 μm are placed onto a sample carrier covered with a special sticky gel. A rotary table transfers the samples to a 3D scanner that scans the sample under an inverted microscope equipped with a digital camera. The sample is analyzed at 120 random positions (sub-samples), covering 30% of the sampled surface. This results in stacked images, each containing 70 two-dimensional images with z differences of 1.5 μm. The system then uses an image recognition algorithm on batch-collected pollen (see https://www.hund.de/en/pollen-monitor for more details) (accessed on 10 February 2022). The classification starts by using different descriptors for the characteristics of the objects (size of the pollen, shape, position and amount of the porus and colpus, structure of the exines, thickness of the intine, structure/form of the plasma). As a result, the device continuously creates tables in csv format, which are locally accessible via the internet at any time. These contain the characteristics of the images as well as the images themselves.

**Figure 1:**
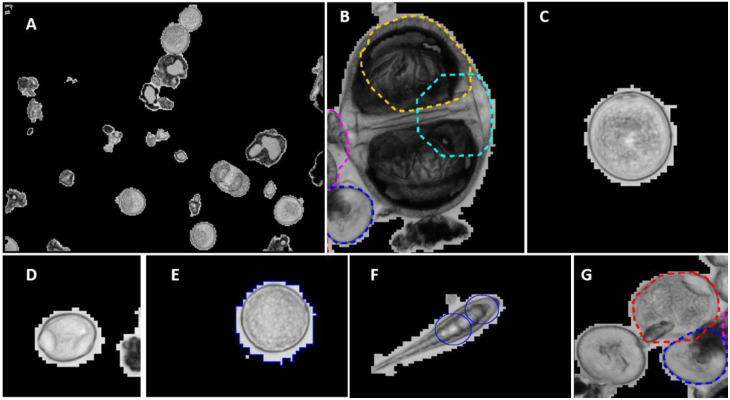
Pollen images by PoMo. (**A**) General shot of photo taken automatically and randomly by PoMo. (**B**) A *Picea* pollen grain, not recognized by PoMo. (**C**) A Poaceae pollen grain. (**D**) An Urticaceae pollen grain. (**E**) A *Plantago* pollen grain. (**F**) An *Alternaria* spore, not recognized by PoMo. (**G**) A *Carpinus* and two *Taxus* pollen grains, not all recognized by PoMo.

The monitoring system was evaluated for its reliability, performance, accuracy and comparability, not only between the two automatic devices, but also against the conventional device measurements. Two years of images contained in spreadsheet files were observed through the Data Analysis Software InfoZoom (HumanIT Software GmbH, Bonn, Germany), from which the experts can see the images taken by the automatic device as well as the pollen type to which each image is attributed and renamed, if necessary, by observing the typical characteristics for pollen types’ recognition (Figure 1).

As performed also by Oteros et al. [26] and Crouzy et al. [14], we applied the following methodological criteria for the evaluation of the automatic pollen monitoring devices.

(A) Reliability: we examined the ability to function reliably with minimal human intervention. Online data availability was calculated as the percentage of time that the instrument reported data on a 3-hourly basis, during almost six consecutive years (29 April 2015–31 December 2020) for the LFU device and almost four years (15 August 2017–31 December 2020) for the IEM device.
(B) Performance and Accuracy: we examined the ability in identifying and counting different pollen types. The capacity of the two PoMo devices to recognise pollen was analysed by comparing with the manual identification, by expert aerobiologists, of a total of 290,743 objects (108,958 from LFU, and 181,785 from IEM) classified as pollen by PoMo itself, during two full pollen seasons (2016 and 2018), and by processing all available data throughout each of these two years. Individual counts produced by each PoMo were manually classified as true positives (TP), false positives (FP), true negatives (TN) and false negatives (FN), following also the standard techniques used to measure performance in binary classification tests [35]. Two different parameters are then taken into account:
  - sensitivity (=TP/(TP + FN)), which refers to actual pollen grains that are correctly identified, and
  - positive predictive value (PPV = TP/(TP + FP), which refers to the proportion of BAA500 identifications that are true.

In addition, during the above validation process of the correct pollen identification by expert aerobiologists, we also attempted to identify and correct ‘Other Pollen types’, so as to potentially improve accuracy in the future.

(C) Comparison of PoMo vs. Hirst-type monitoring technique: Apart from comparing the abundances of pollen per taxon and per device, as conducted by Oteros et al. [26], we additionally assessed the pollen seasonality per taxon and per device. We evaluated the onset, peak, end and duration of the pollen seasons, using the Nilsson and Persson [36] method for defining the main pollen seasons. This method includes 95% of seasonal total pollen concentration, starting on the day that 2.5% of the total pollen was recorded and ending on the day that 97.5% of the total pollen was registered. To identify potential significant relationships between the measurements from the PoMo and the Hirst-type monitoring system, we also used General Linear Models (GLM) and linear regressions for the pollen season phenological traits, namely, the start, peak and end dates, along with the season duration, as well as the Annual Pollen Integral and the annual peak concentration.
(D) Other particle types: we examined the ability of the automatic device to efficiently identify and count other particles than pollen, such as airborne fungal spores. To achieve this, we manually classified more than 50,000 objects originally identified by the automatic PoMo in LFU either as ‘Spores’ (fungal spores) or as ‘NoPollen’ (neither pollen, spores nor any known bioaerosol type), during the expected main spore season, namely May to August 2016.

## 3. Results

### 3.1. Reliability

**Figure 2:**
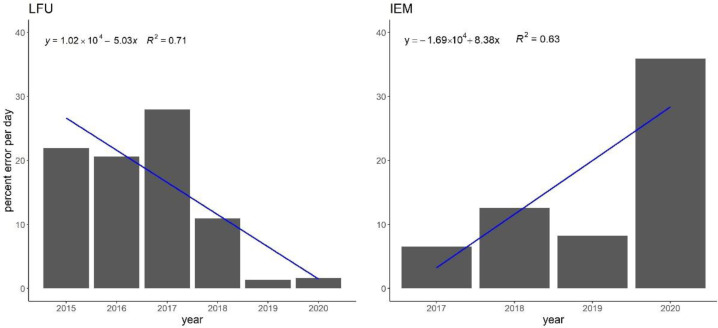
PoMo reliability in two sites, LFU (2015–2020; **left**) and IEM (2017–2020; **right**). The number of cases with data gaps (in %) are shown.

Reliability of both devices is shown in Figure 2. Both seem to have significantly fewer errors over time; however, the IEM device during the year 2020 seems to be highly erroneous. Within the main pollen season, the data flow interruptions seem low (5–10% on average), but also seem to be steady over time, without improvement. On the other hand, sampling has been interrupted by full-day failures on a consistent average of more than 10% for the majority of sampling years (Figure 2, right panel). In Table 1, one may see that a median of 17–19 days of whole-day gaps was observed for both PoMo devices, within the main pollen season of the 15 most abundant pollen types in Augsburg, Germany.

### 3.2. Classification Performance

**Figure 3:**
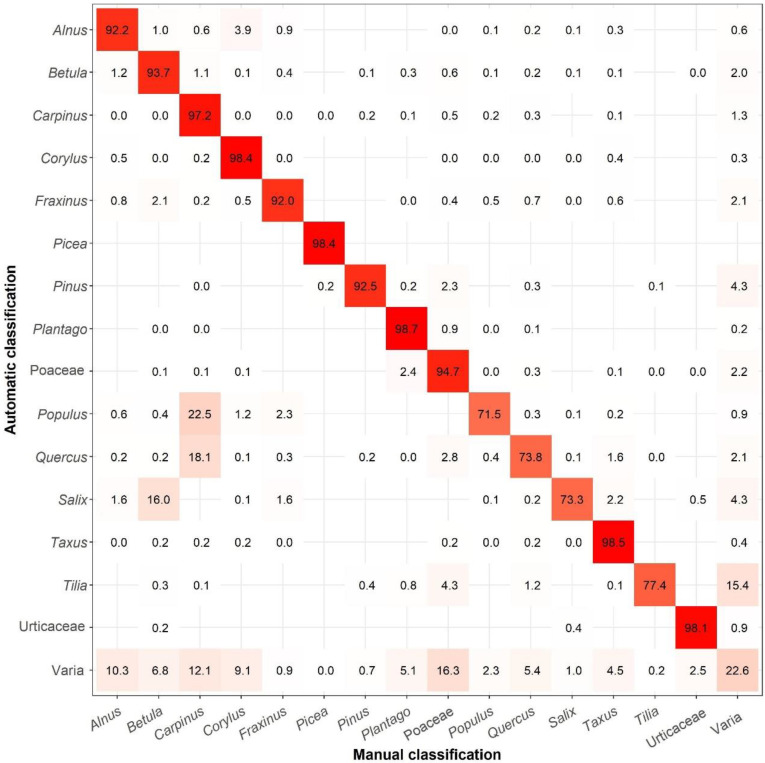
Cross-classification table (confusion matrix) between the predicted pollen values (automatic classified) and target values (manually classified) in LFU PoMo. Empty nodes indicate that there is no confusion between these pollen types.

From the LFU site, we checked 993,081 objects, of which 145,699 were automatically classified by PoMo as pollen from different taxa. These objects were automatically labelled into 38 different pollen types and also into “Varia” (which refers to unknown pollen types), when the PoMo algorithm could not classify them in one of the known pollen types. As is shown in Figure 3, from all the pollen classes identified by LFU PoMo (the 15 most abundant pollen types, along with ‘Varia’), the sampler had an overall accuracy of 85.9%. This accuracy ranges from less than 80% for *Populus*, *Quercus*, *Salix* and *Tilia*, and up to more than 98% for *Corylus*, *Picea*, *Plantago*, *Taxus* and Urticaceae (Figure 3). In the least accurate cases, PoMo confuses *Populus*, *Quercus* and *Salix* mostly with *Betula* and *Carpinus*, whereas it cannot identify *Tilia* frequently and classifies this as ‘Varia’ (Figure 3). The actual class of ‘Varia’ was only true by 22.6%, and the rest were correctly identified mostly as *Alnus*, *Carpinus* and Poaceae (Figure 3). 

**Figure 4:**
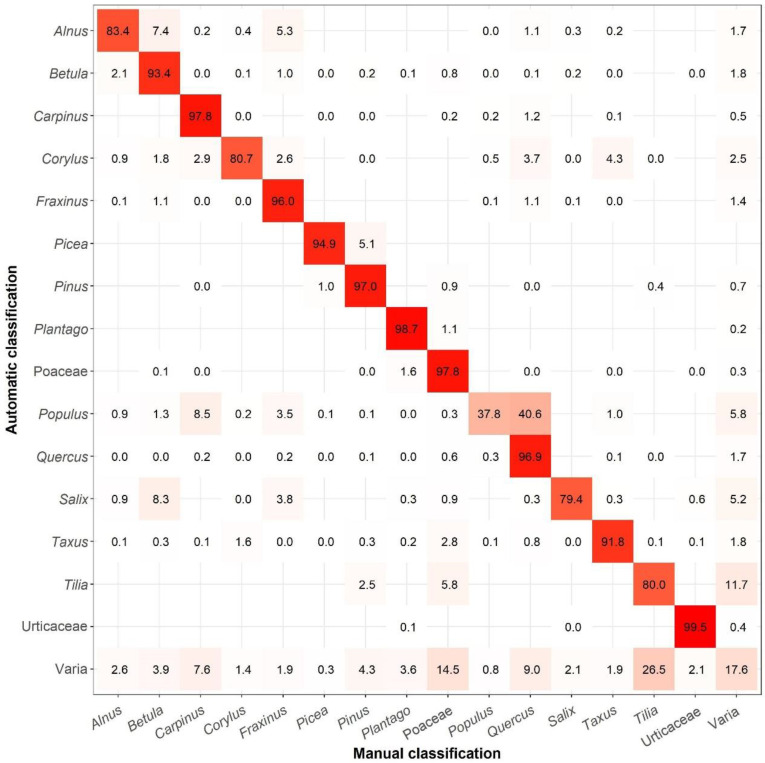
Cross-classification table (Confusion matrix) between the predicted pollen values (automatic classified) and target values (manually classified) in IEM PoMo. Empty nodes indicate that there is no confusion between these pollen types.

Figure 4 summarises the corresponding accuracy findings for the IEM PoMo. In this (Figure 4), the BAA500 recognized 1,465,413 objects, of which 181,785 were classified like pollen from different species. The accuracy range varies from less than 40% for *Populus*, and less than or equal to 80% for *Salix* and *Tilia*, up to more than 98% for *Plantago* and Urticaceae; The overall accuracy reached on average 83.9% for all the 15 most abundant and ‘Varia’ pollen types. In the least accurate cases, PoMo very largely confuses *Populus* with *Quercus*; it also confuses *Salix* with *Betula*, whereas *Tilia* frequently cannot be identified once more and is it classified as ‘Varia’ (Figure 4), as also is the exact case for LFU PoMo. The actual class of ‘Varia’ was only true by 17.6%, and the rest were correctly identified mostly as *Carpinus*, Poaceae, *Quercus* and *Tilia* (Figure 4).

**Figure 5:**
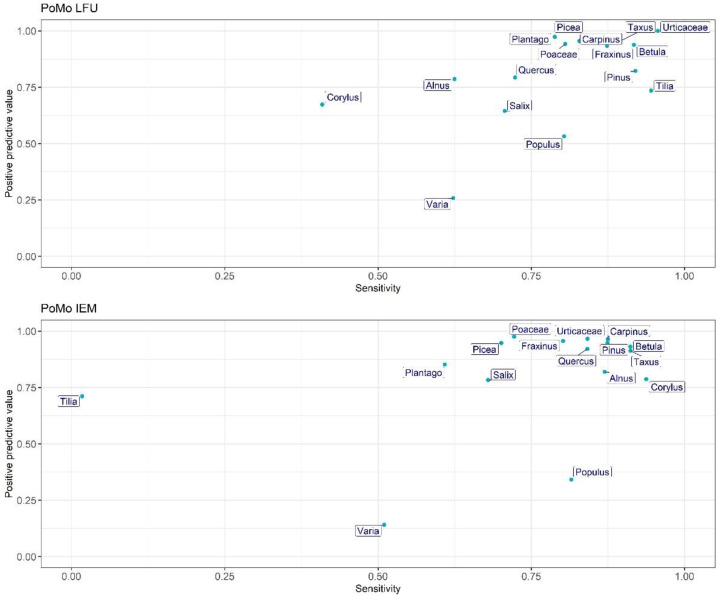
PoMo capability of identifying different pollen types. Sensitivity in the *X*-axis shows the percentage of correctly identified particles. The positive predictive value (PPV) in the *Y*-axis shows the ratio of automatic identifications that were correct.

Figure 5 summarises the performance of each of the PoMo systems in classifying each pollen type for the 15 most abundant taxa. The majority of pollen taxa are positioned in the upper right corner of the graph for both devices, representative of optimum performance, of approximately more than 80%. However, it appears that *Populus* and *Tilia* are incorrectly non- or mis-classified consistently in both devices (Figure 4). Interestingly, the LFU PoMo also only seems to have problems classifying *Corylus* correctly, as well as *Salix*; likewise, the sensitivity of the two PoMo systems differs greatly for the same taxa, namely *Corylus* and *Tilia*, which seem to work perfectly for the one, but not for the other (Figure 5).

**Figure 6:**
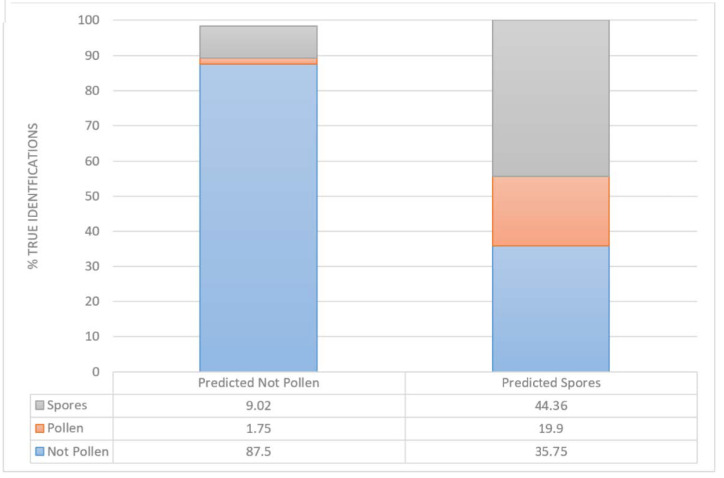
Bar chart of the accuracy of classification for all objects. The number of total objects classified automatically vs. manually is given, identifying Pollen, Spores and Not Pollen (i.e., anything else other than the above two categories).

To be able also to evaluate the identification accuracy of other air particles, a total of 58,200 objects identified by the automatic algorithm of the PoMo device as “No Pollen” and “Spores” was manually classified. Of all the checked objects, 87% were correctly classified as “No Pollen”. However, about 9% were mistakenly classified as “No Pollen” while they were actual “Spores”, and another 1.7% was pollen (Figure 6). This highlights that the aforementioned accuracy percentages are to an extent overestimated.

### 3.3. Comparisons of Automatic vs. Conventional Pollen Measurements

**Figure 7:**
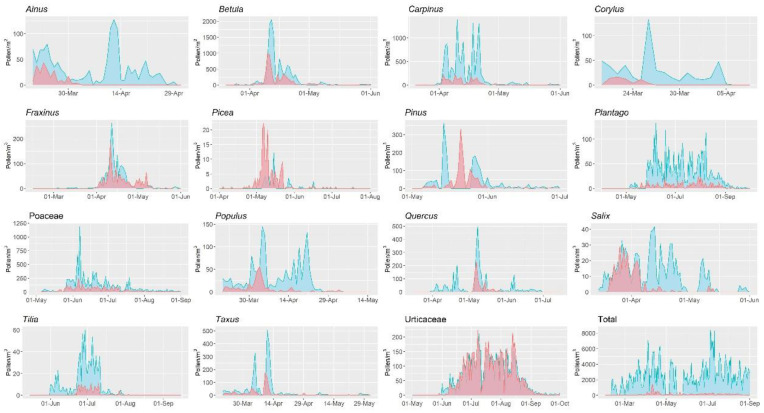
Seasonality of pollen concentrations monitored by the LFU PoMo (blue) and the Hirst-type system (red) for the 15 most abundant pollen types, and their total pollen load, in Augsburg.

**Figure 8:**
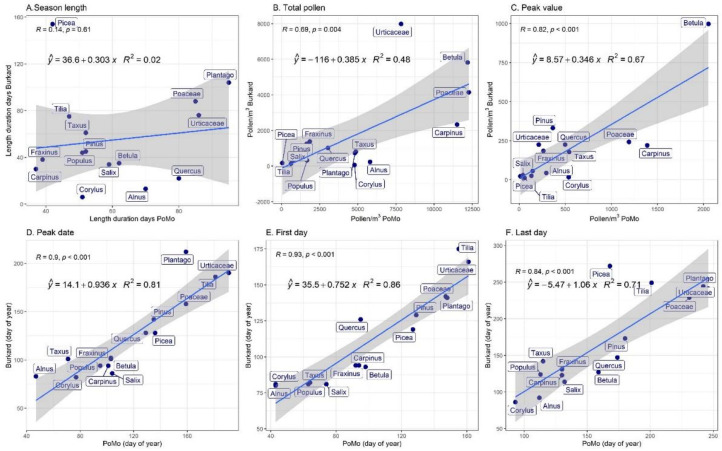
Linear regressions (blue lines) between LFU PoMo and Hirst-type main pollen season traits are shown, with 95% confidence intervals (grey area). The coefficient of determination (*R*^2^) and Pearson’s correlation coefficient (*r*) are also shown. (**A**) The pollen season length (duration between the first and the last pollen day). (**B**) The Annual Pollen Integral (cumulative pollen concentration per year). (**C**) Peak daily pollen concentration (maximum concentration per year). (**D**) Date on which the maximum concentration per year was observed. (**E**) The date on which the first pollen was observed in the year. (**F**) The date on which the last pollen was observed in the year.

**Figure 9:**
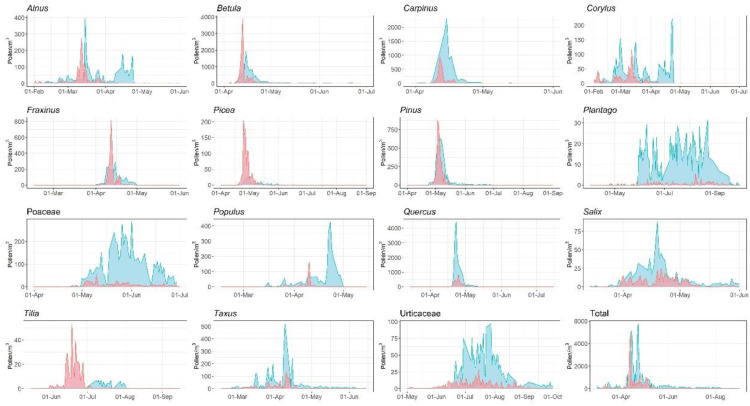
Seasonality of pollen concentrations monitored by the IEM PoMo (blue) and the Hirst-type system (red) for the 15 most abundant pollen types, and their total pollen load, in Augsburg.

**Figure 10:**
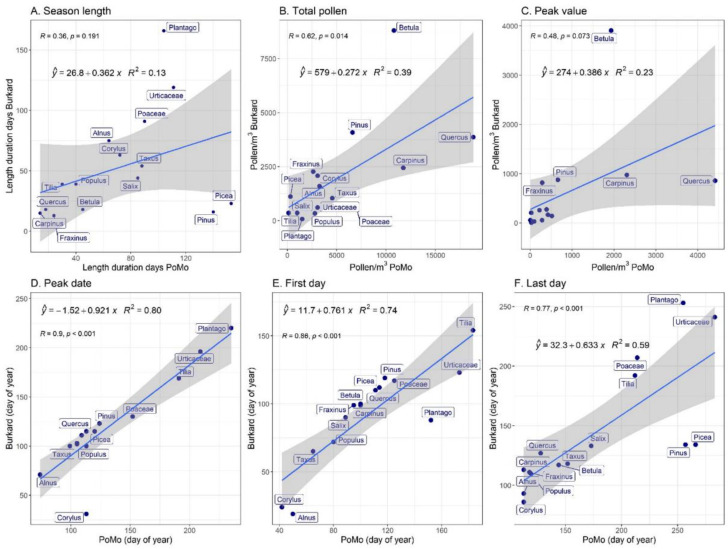
Linear regressions (blue lines) between IEM PoMo and Hirst-type main pollen season traits are shown, with 95% confidence intervals (grey area). The coefficient of determination (*R*^2^) and Pearson’s correlation coefficient (*r*) are also shown. (**A**) The pollen season length (duration between the first and the last pollen day). (**B**) The Annual Pollen Integral (cumulative pollen concentration per year). (**C**) Peak daily pollen concentration (maximum concentration per year). (**D**) Date on which the maximum concentration per year was observed. (**E**) The date on which the first pollen was observed in the year. (**F**) The date on which the last pollen was observed in the year.

To evaluate the comparability of each PoMo device, we analysed its measurements against those of the Hirst-type next to each, for both LFU PoMo (Figure 7 and Figure 8) and IEM PoMo (Figure 9 and Figure 10). Regarding the overall seasonality of each pollen type, as displayed in Figure 7 and Figure 9, it may be concluded that for the majority of taxa (of the 15 most abundant in Augsburg) the seasons have major differences, as monitored by the PoMo and the Hirst-type devices, for both sites. In both, the majority of taxa exhibit at least a two-fold higher concentration in the PoMo measurements compared to the Hirst-type; exceptions seem to be *Betula*, *Fraxinus* and Urticaceae in LFU (Figure 7), and *Picea* in both sites, which remarkably is the only example to show lower concentrations in both PoMo measurements (Figure 7, Figure 8 and Figure 9). Likewise, there seems to be low comparability for the pollen season attributes (onset, peak, end, duration) of both PoMo devices when compared to the respective of the nearby Hirst-type sampler: the latter, in most pollen types, seems to fail to catch an earlier onset, which only the PoMo (in both sites) manages to capture (Figure 7, Figure 8 and Figure 9). 

On the other hand, when comparing the measurements of the paired devices, Hirst-type and PoMo per site, as a whole there is usually a significant correlation, confirming thus the comparability of the monitoring systems (Figure 8, Figure 9 and Figure 10). In Figure 8 and Figure 10, the relationship between abundance traits is given (Annual Pollen Integral (Figure 8B and Figure 10B) and Peak Value (Figure 8C and Figure 10C)), as well as seasonality attributes, such as season length (Figure 8A and Figure 10A), season peak date (Figure 8D and Figure 10D), and season start and end (Figure 8E,F and Figure 10E,F, respectively). As also indicated in Figure 7 and Figure 9, it is obvious that the Hirst-type device consistently underestimates the pollen concentrations, in terms of both annual integral as well as peak value per season (note the different axes in Figure 8B,C, and Figure 10B,C). For season occurrence, the relationships between the two samples are better, often reaching an average of 80%; however, there are taxon-specific differences; for this reason, in the comparisons in both sites, the season length never correlates significantly, showing the non-comparability in long season tails at the start and the end of the seasons in many pollen types (Figure 7, Figure 8A, Figure 9 and Figure 10A). These results indicate that both air samplers have different abilities in capturing particles, since they showed different and variable values in the main parameter considered.

The above results refer to the originally classified pollen concentrations of both PoMo devices. If one compares the same Hirst-type measurements with the manually checked and classified PoMo measurements, the results are as shown in Figures S1–S4. Figures S1 and S3 show that the seasonality and its shape per taxon have not dramatically changed. For this reason, Figures S2 and S4 also seem similar to the ones discussed above. An exception in the LFU PoMo is the season attribute of the last day (Figure S2F), where the relationship became stronger with the manually classified data, increasing from 0.71 to 0.90, a fact that implies the loss of some season tails from the original PoMo classifications. Likewise, for the IEM PoMo, the annual pollen integral relationship between the two monitoring systems increased from 0.39 to 0.55 (Figure S4B), the peak date relationship from 0.80 to 0.95 (Figure S4D), and the first day occurrence relationship from 0.74 to 0.89; these also indicate that, after manual classification, early and high incidents had now been identified and the performance of the IEM PoMo improved.

Summarising the findings from Figure 7, Figure 8, Figure 9 and Figure 10, it is seen that the pollen season as measured by PoMo started later than the Hirst-type by 3 days (median), peaked later by a median of 5 days and ended later by 14 days. However, regarding the pollen abundances observed in the different monitoring systems, results were less comparable, with the PoMo pollen season showing higher daily peak concentrations than the Hirst-type by 66 pollen grains per cubic metre of air, while the annual pollen integral was much higher in the PoMo pollen season by 2080 pollen when compared to the Hirst-type monitoring system.

## 4. Discussion

Automatic bioaerosol monitoring has been a hot research topic for a variety of scientific disciplines during the last decade, for aerobiologists, ecologists, bioclimatologists, mathematical modelers, engineers, and medical doctors. Much progress has been made as, for example, in the cases of O’Connor et al. [37] with a WIBS-4 device or of Crouzy et al. [14] with Plair PA-300 in Switzerland [38,39], but the issues of reliability and replicability of results are still under investigation or dispute. There have been recently initiated major inter-comparison campaigns via European projects [40,41], as the ‘Autopollen’ within the framework of EUMETNET (https://www.eumetnet.eu/activities/miscellaneous/current-activities-mi/autopollen/; accessed on 15 February 2022), as well as the EU-COST Action CA18226 ‘ADOPT’ (https://www.cost.eu/actions/CA18226/; accessed on 15 February 2022). However, to date, there is no conclusive published information on intercomparison campaigns among automatic devices for differing environmental regimes, between different devices of the same brand, and against paired comparisons with the ‘gold-standard’ conventional Hirst-type monitoring system. Regarding the current study’s automatic system, Oteros et al. [26] carried out such an intercomparison, evaluating its reliability and performance, with reportedly exceptional results. Nonetheless, in the work presented here, and having tested 15 different pollen morphotypes and in two different sites, we observed that there is an overall good performance, but still with reliability and accuracy issues raised for specific pollen types. To efficiently address these concerns, we followed a four-step procedure in the evaluation process: we tested the automatic system for its: (1) reliability in providing uninterrupted data, (2) performance and accuracy in correctly and adequately identifying all objects based on its image recognition algorithm, (3) comparability against a similar system at another site, and against the conventional Hirst-type sampler in both of the same sites, and (4) ability to distinguish between different air particles, mostly fungal spores.

Regarding the reliability of the instrument, in one of the locations (LFU), the device was online for about 75% of the time on average, in most cases because of breakdowns (more than 7 full days with no data, noticeably within the main pollen season). The reliability of the BAA500 increased with time. However, the device located in the other location showed different results with important breakdown situations during the 2020 pollen season. The Hirst volumetric spore trap revealed a reliability in approximately 100% of the time monitored. However, the workload required for sampling with the Hirst-type method is high, forcing many monitoring stations to work during the main flowering season only (https://ean.polleninfo.eu/) (accessed on 10 January 2022).

Regarding the identification and measuring of different pollen types, most of the pollen taxa were recognised in the majority of cases. However, the automatic PoMo was unable to identify some pollen types, usually consistently between the devices, i.e., *Populus* and *Salix*. Whenever discrepancies were found in the manual vs. automatic classification of the automatic system, this was due to shortcomings or flaws in the algorithm of the identification: for example, significantly lower *Tilia* pollen concentrations could be explained because of filters applied to define the main pollen season per pollen type. This is actually true for the majority of pollen types, which practically means that earlier occurrence of winter pollen seasons is prone to be missed; the only solution to this is to reconstruct the algorithm to omit such filters. Moreover, there were also differences in misclassification levels between the two sites (e.g., *Quercus* and *Populus*), of almost 30% in some cases between both locations. One would expect such differences in only very diverse environments in terms of bioclimate and vegetation, when potentially the training dataset would not be large enough. However, in our case here we refer to the same city, of average size and common vegetation, which actually suggests differences or shortcomings in the software or hardware of the different automatic devices. In such similar environments, one would also expect similar performance and accuracy levels, which raises questions as to whether all commercial units are synchronised in terms of manufacturing quality and algorithm updates.

Likewise, when it comes to identifying other bioaerosol types, namely fungal spores, the automatic system seems unable to perform efficiently. One of the main reasons seems to be that already highlighted by Schiele et al. [42], viz. the cropping technique of acquired images is not accurate: cropping areas for image recognition are considered *de facto* to be round, so as to be efficient for the identification of the mostly round pollen grains, but then this filter is definitely not true for many other particles, including fungal spores, or deformed or broken pollen. 

On the other hand, the airborne pollen levels recorded by the Hirst-type system were significantly lower than those reported by the automatic PoMo in our study for the majority of taxa examined. While the seasonality seems more comparable, there are still mismatches in specific taxa, with the conventional Hirst-type system often missing long tails towards the end of the seasons, sometimes with a difference of several weeks. While it cannot be conclusively decided which of the devices provides the optimal observations, this raises questions as to which is the genuine pollen exposure, viz. the one most relevant clinically.

Slightly significant correlations were obtained for pollen concentrations collected by both samplers, the automatic PoMo and the conventional Hirst-type; in both sites, the relationships were significant but weak, unlike the very strong ones that Oteros et al. [26] detected. This indicates a dissimilar capacity to capture particles, which could be explained due to the fact that the system is not the same; the air flow by the novel PoMo is higher and operates on an intermittent 3-hourly air flow, in contrast to the continuous, but lower, air flow of the Hirst-type sampler. Moreover, they might have differences in their ability to collect particles depending on their aerodynamic features. Although most pollen types showed a significant correlation in their abundances between the two sampling systems (i.e., *Fraxinus*, *Pinus*, Urticaceae), most were trapped in different quantities. These correlations improve when manual classification is performed, but the correlation coefficient in any case does not exceed 0.60. These differences in concentration by both samplers should be considered when comparing results, given also that the difference is of a remarkable two-fold magnitude at least.

Regarding pollen seasonality, the main pollen season seems to coincide in most of the pollen types (regardless of the abundance). The relationships in seasonal traits (start, peak, end of main pollen season) become stronger after manual classification of the automatic PoMo, but the length of the season does not always correlate between the two monitoring systems. Having said this, such differences were to be expected, as they were also reported in previous studies with Hirst-type samplers, even when located next to each other [43,44]. The important question raised here is which pollen season length (and corresponding first start and full end of the overall season) is the most relevant clinically.

Automatic identification systems could be the solution for pollen monitoring if they would suppose the same constant error, since Hirst-type traps have showed human variability in counting [45]. However, our results showed that the accuracy and sensitivity for some pollen types between two PoMo devices could vary even with the same recognition algorithm. In addition, the difference in pollen concentration collected with respect to the ‘gold-standard’ method must be established in order to compare results. However, despite some observed differences, the novel technological advancements in bioaerosol monitoring exhibit certain advantages: online, automatic, real-time measurements, significantly reduced human workload (with improved recognition) and stable methodology, as well as high recognition ability for most pollen types. The improvement of the methodology and extension to more taxa is beyond the possibility of this study. Currently there are several advances in this type of technology, and they are changing rapidly. This kind of study is essential to address the prospects of generalization and replicability of new pollen monitoring systems. While the present study certainly exhibits prominent strengths, such as the uniqueness and large size of the training dataset, as well as the investigation of the full diversity of airborne pollen taxa, it also displays some limitations. Because of the repeated data gaps per year, even more years of data would be invaluable for safer conclusions. Moreover, as a future prospect, manual classification of the full diversity of fungi would allow us to improve and hopefully establish also a fully operational automatic monitoring system for this type of bioaerosol. Standardising and generalizing, potentially for wider uses and applications in the field, a robust and highly accurate identification algorithm for the majority of allergenic pollen and spore taxa would be a highlight in future research. 

The new generation of automated and real-time pollen monitoring systems is expected to serve a very important societal purpose: they will comprise the cornerstone of first-line prevention against allergic diseases, but also for emerging health risks like viral infections. Living currently in the COVID-19 pandemic era, real-time information on airborne pollen concentrations will also contribute to the confrontation with major viral spreading, as has been reported by Damialis et al. [7]: airborne pollen concentrations are positively correlated with increased SARS-CoV-2 infection rates, and this makes real-time pollen measurements more important than ever. If we additionally consider the dramatic impacts of the ongoing climate change on pollen seasons, shifting them earlier within the late winter viruses’ seasons and increasing their intensity [6,46], the development of operational systems of automatic pollen monitoring seems urgent and with multiple potential applications and health benefits.

## 5. Conclusions

The current study provides results from an extensive monitoring campaign of two paired systems in two sites, in Augsburg, Germany, since 2015. The salient findings were that the novel automatic device still has reliability issues, exhibiting monitoring (data) gaps of a median of 17–19 days within the main pollen season, but also already displays a performance average of 85%. While there are some pollen types that the automatic system cannot accurately identify, it seems, on the other hand, to perform better compared to the conventional, ‘gold-standard’ Hirst-type monitoring system: the Hirst-type pollen abundances were consistently lower, and with frequently shorter season lengths. Given that we compare here a 70-year-old technology against a decade-old one, it is evident that the novel automatic pollen monitoring has already shown already great potential and important signs of efficiency and accuracy, despite its young age. Reliability issues that still exist are definitely a disadvantage, but we anticipate that this new generation of automated bioaerosol monitoring systems will soon change the aerobiological era, as known for decades now. Given the dramatic differences in the abundance and onset, end and duration of the pollen season for a wide variety of pollen types, this will most probably be reflected in the accuracy of pollen season occurrence and intensity forecasting. This will be an important step towards the most efficient form of allergy management via real-time pollen and fungal spores’ measurements.

## Figures and Tables

**Table 1:** Number of days with complete failures (days without data) in the LFU and IEM PoMo, for the 15 most abundant pollen types in Augsburg (>0.5% of Annual Pollen Integral), and the median per site (of all the years of operation per PoMo).

Pollen Type	LFU Gaps (Days)	IEM Gaps (Days)
***Alnus***	1	54
***Betula***	23	31
***Carpinus***	17	18
***Corylus***	0	47
***Fraxinus***	17	10
***Picea***	20	19
***Pinus***	15	10
***Plantago***	18	25
**Poaceae**	30	26
***Populus***	9	14
***Quercus***	14	8
***Salix***	2	15
***Taxus***	17	28
***Tilia***	3	10
**Urticaceae**	19	44
**MEDIAN**	**17**	**19**

## Data Availability

Data may be become available upon reasonable request.

## Supplementary Materials

The following supporting information can be downloaded at: https://www.mdpi.com/article/10.3390/ijerph19042471/s1, Figure S1: Seasonality of pollen concentrations monitored by the LFU PoMo (blue colour), after manually classified, and the Hirst-type system (red colour), for the 15 most abundant pollen types, and their total pollen load, in Augsburg; Figure S2: Linear regressions (blue lines) between LFU PoMo and Hirst-type main pollen season traits are shown, with 95% confidence intervals (grey area). The coefficient of determination (R^2^) and Pearson’s correlation coefficient (r) are also shown; Figure S3: Seasonality of pollen concentrations monitored by the IEM PoMo (blue colour), after manually classified, and the Hirst-type system (red colour), for the 15 most abundant pollen types, and their total pollen load, in Augsburg; Figure S4: Linear regressions (blue lines) between IEM PoMo and Hirst-type main pollen season traits are shown, with 95% confidence intervals (grey area). The coefficient of determination (R^2^) and Pearson’s correlation coefficient (r) are also shown.
