# Supplementary material for: Detecting Airborne Pollen Using an Automatic, Real-Time Monitoring System: Evidence from Two Sites
Source: Int J Environ Res Public Health. 2022 Feb 21;19(4):2471. doi: 10.3390/ijerph19042471 (PMC8872361; doi:10.3390/ijerph19042471)
Supplement: Supplementary file 1 [file ijerph-19-02471-s001.zip › SUPPLEMENTARY MATERIAL LEGENDS.pdf]

SUPPLEMENTARY MATERIAL LEGENDS

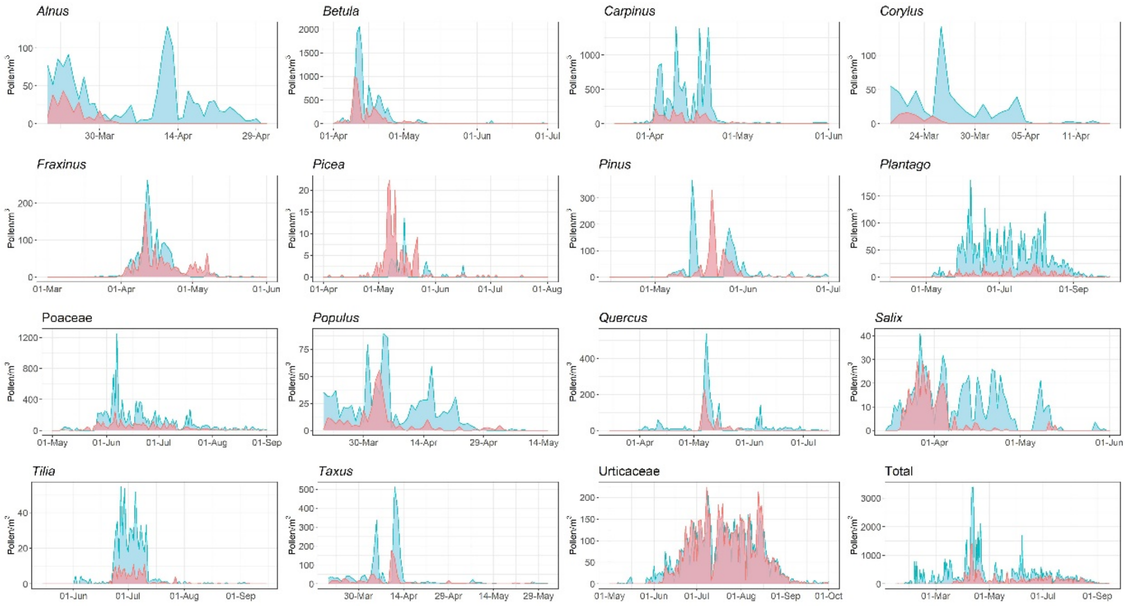

Figure S1. Seasonality of pollen concentrations monitored by the LFU PoMo (blue colour), after manually classified, and the Hirst-type system (red colour), for the 15 most abundant pollen types, and their total pollen load, in Augsburg.

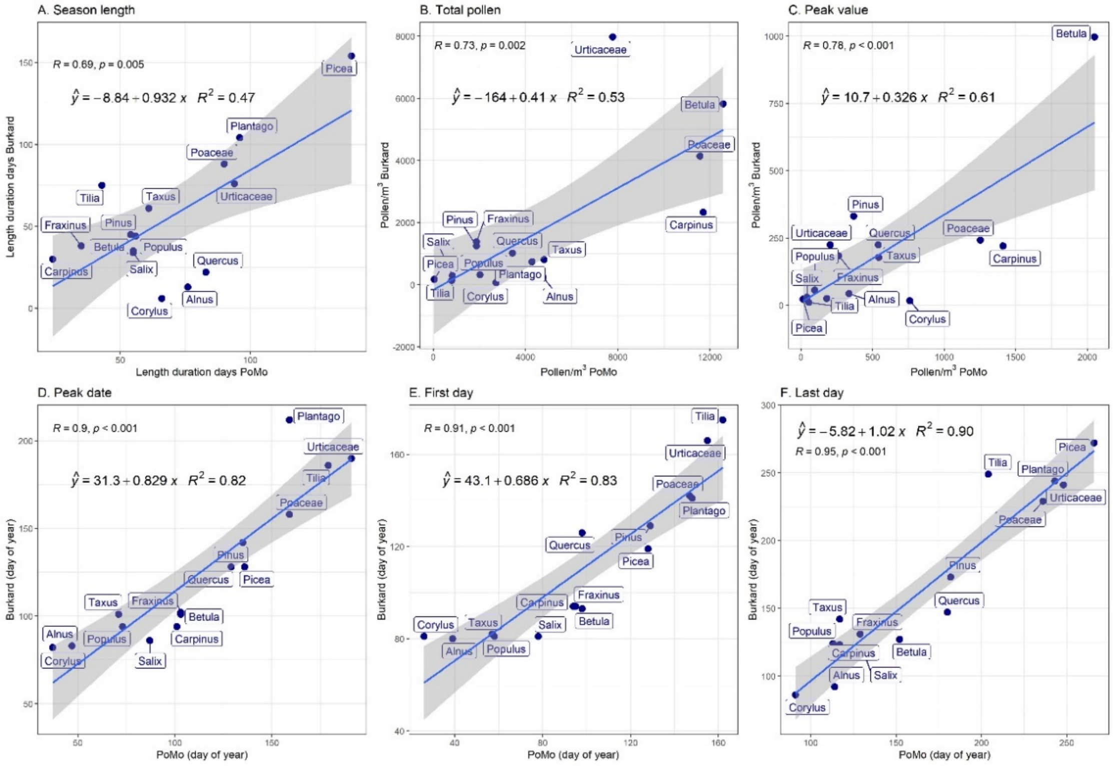

Figure S2. Linear regressions (blue lines) between LFU PoMo, after manually classified, and Hirst-type main pollen season traits are shown, with 95% confidence intervals (grey area). The coefficient of determination (R^2) and Pearson's correlation coefficient (r) are also shown.

- **A.** The pollen season length (duration between the first and the last pollen day)
- **B.** The Annual Pollen Integral (cumulative pollen concentration per year)
- **C.** Peak daily pollen concentration (maximum concentration per year)
- **D.** Date on which the maximum concentration per year was observed
- **E.** The date on which the first pollen was observed in the year
- **F.** The date on which the last pollen was observed in the year

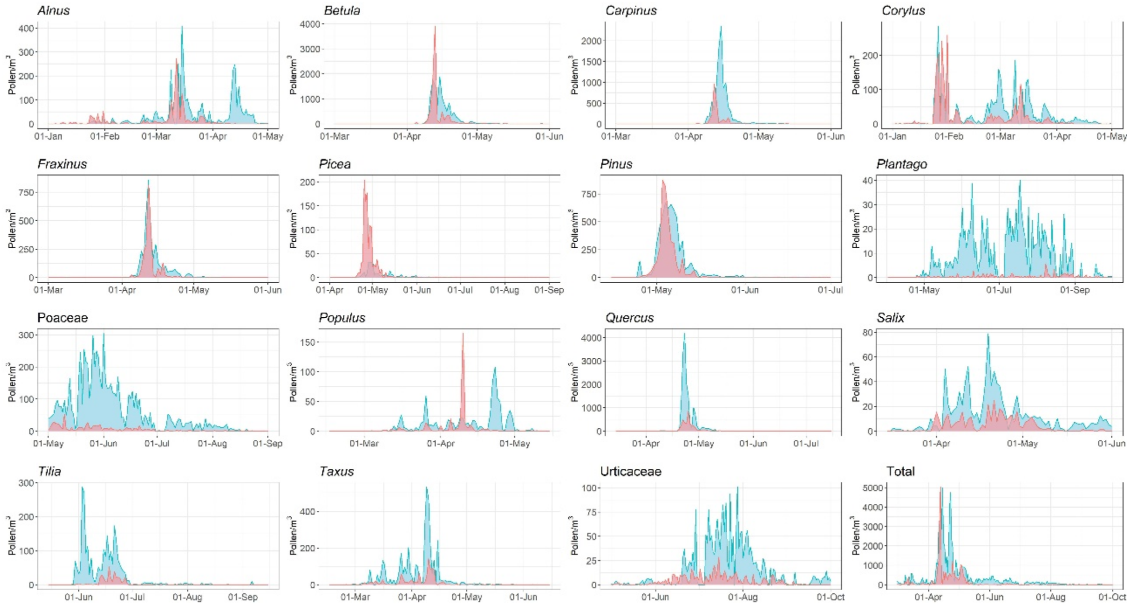

Figure S3. Seasonality of pollen concentrations monitored by the IEM PoMo (blue colour), after manually classified, and the Hirst-type system (red colour), for the 15 most abundant pollen types, and their total pollen load, in Augsburg.

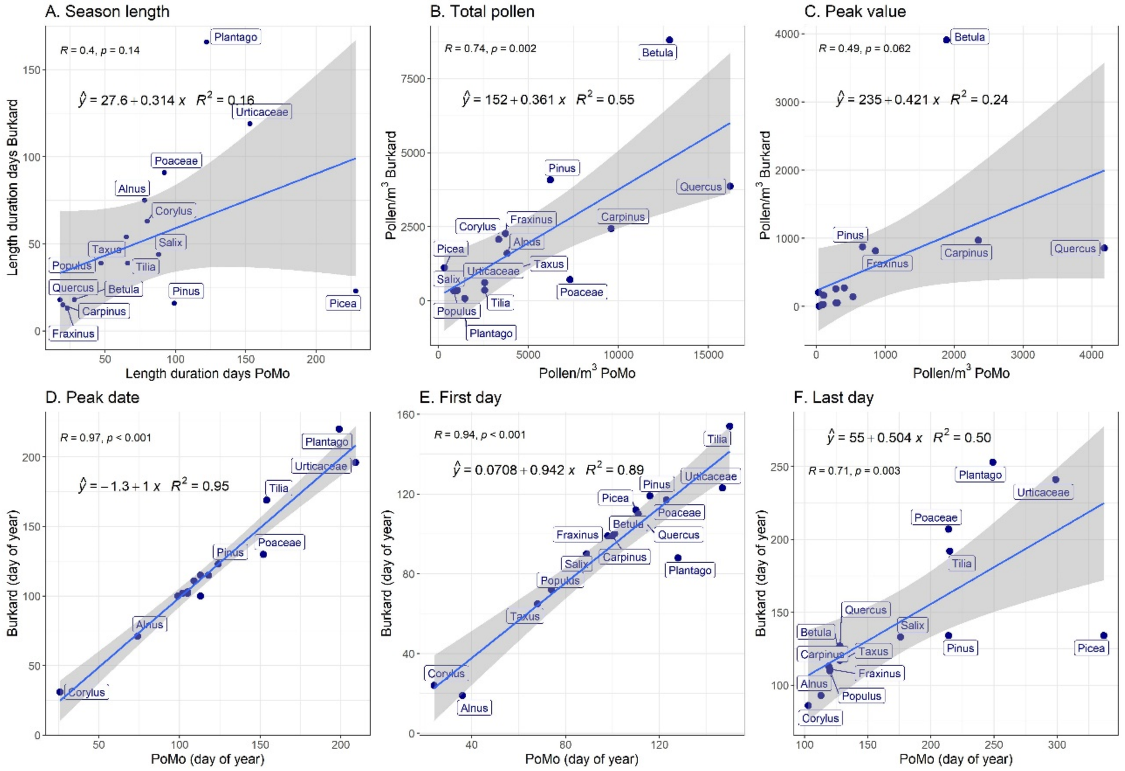

Figure S4. Linear regressions (blue lines) between IEM PoMo, after manually classified, and Hirst-type main pollen season traits are shown, with 95% confidence intervals (grey area). The coefficient of determination (R^2) and Pearson's correlation coefficient (r) are also shown.

- **A.** The pollen season length (duration between the first and the last pollen day)
- **B.** The Annual Pollen Integral (cumulative pollen concentration per year)
- **C.** Peak daily pollen concentration (maximum concentration per year)
- **D.** Date on which the maximum concentration per year was observed
- **E.** The date on which the first pollen was observed in the year
- **F.** The date on which the last pollen was observed in the year
